# Tetra­aqua­bis­(pyridine-3-carbo­nitrile-κ*N* ^1^)nickel(II) benzene-1,4-di­carboxyl­ate tetra­hydrate

**DOI:** 10.1107/S2056989020015832

**Published:** 2021-01-01

**Authors:** Monsumi Gogoi, Birinchi Kumar Das

**Affiliations:** a Department of Chemistry, Gauhati University, Guwahati-781014, Assam, India; b Bhattadev University, Bajali, Pathsala-781325, Assam, India

**Keywords:** Ni^II^ complex, terephthalate, pyridine-3-carbo­nitrile, π–π inter­action, graph set, crystal structure

## Abstract

A nickel(II) terephthalate complex, *viz.* [Ni(C_6_H_4_N_2_)_2_(H_2_O)_4_](O_2_CC_6_H_4_CO_2_)·4H_2_O, has been synthesized and studied by single-crystal X-ray diffraction. It crystallizes in the triclinic space group *P* . The crystal structure shows an approximately octa­hedral coordination environment of the complex with the [Ni(H_2_O)_4_(3-NCpy)_2_]^2+^ (3-NCpy is pyridine-3-carbo­nitrile) cation associated with four free water mol­ecules and hydrogen bonded to a terephthalate dianion [graph set *R* ^2^_2_(8)]. The supra­molecular structure of the compound is stabilized by a three-dimensional array of O—H⋯O and O—H⋯N hydrogen bonds, along with π–π stacked pyridine-3-carbo­nitrile rings and C—H⋯O inter­actions.

## Abstract 2

The structure of a nickel(II) terephthalate complex, *viz.* tetra­aqua­bis­(pyridine-3-carbo­nitrile)­nickel(II) benzene-1,4-di­carboxyl­ate tetra­hydrate is described.

## Chemical context   

Multi-carboxyl­ate ligands with suitable spacers, especially benzene-multi­carboxyl­ate ligands, are frequent choices for coordination chemistry as they feature a broad range of coordination modes and can result in the formation of systems with variable complexity ranging from mol­ecular complexes to metal–organic frameworks of different dimensionality (Janiak & Vieth, 2010 ▸; Kim *et al.*, 2001 ▸). Benzene-1,4-di­carboxyl­ate (terephthalate) ligands have received increased attention in the field of coordination chemistry, especially as building blocks for coordination polymers, mainly with porous networks with varied metal ions (Kim *et al.*, 2003 ▸). As a result of the presence of conjugation, the terephthalate anion can provide an electronic pathway for delocalization of electrons belonging to the *d-*orbitals of the metal ion, thus changing its magnetic properties. The most important factor that affects magnetic exchange pathways between two metal centres is the proper choice of bridging ligands since they influence the magnetic strength and behaviour of the mol­ecule (Massoud *et al.*, 2006 ▸; Mukherjee *et al.*, 2003 ▸; Rogan *et al.*, 2000 ▸). Coordinated ligand systems containing electron-donor as well as acceptor sites also give rise to metallo­supra­molecular assemblies. Hence, pyridine-3-carbo­nitrile (3-NCpy) with the electron-withdrawing nitrile group as the acceptor along with the pyridyl nitro­gen atom as the donor stands as a suitable ligand in this regard. Despite the availability of two potentially coordinating sites, not many compounds having pyridine-3-carbo­nitrile as a bidentate bridging ligand are known (Heine *et al.*, 2018 ▸). The nitrile group may also be expected to take part in hydrogen bonding and π–π inter­actions. In this work, we describe our results on the synthesis and crystal structure of a pyridine-3-carbo­nitrile-based Ni^II^–terephthalate complex, *viz*. [Ni(H_2_O)_4_(3-NCpy)_2_][O_2_CC_6_H_4_CO_2_]·4H_2_O.
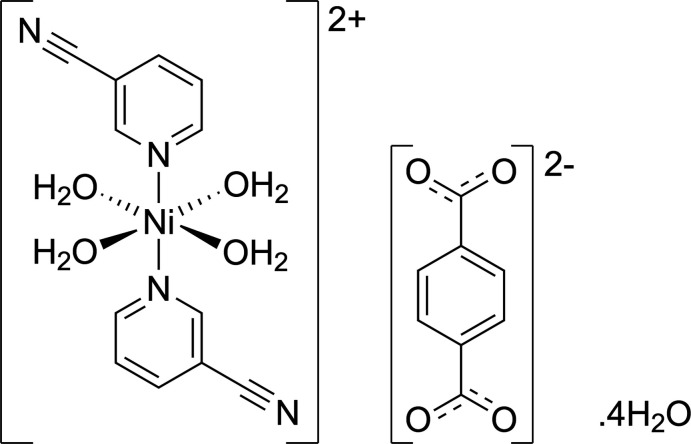


## Structural commentary   

**Figure 1:**
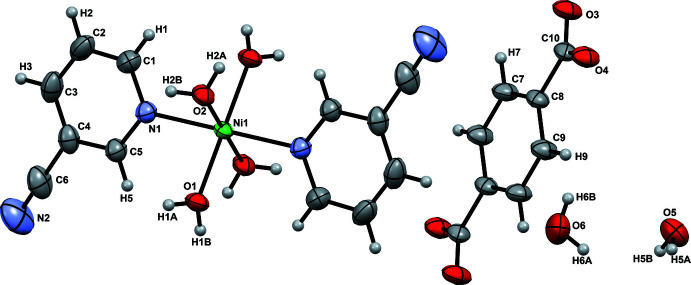
*ORTEP* diagram of [Ni(H_2_O)_4_(3-NCpy)_2_][O_2_CC_6_H_4_CO_2_]·4H_2_O showing the atom-labelling scheme (ellipsoids drawn at the 50% probability level; unlabelled atoms generated by the symmetry operations 2 − *x*, 2 − *y*, −*z* for the cation and 1 − *x*, 1 − *y*, 1 − *z* for the anion).

The title compound, [Ni(H_2_O)_4_(3-NCpy)_2_][O_2_CC_6_H_4_CO_2_]·4H_2_O is a discrete coordination complex and it crystallizes in the triclinic system with space group *P* 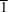. An *ORTEP* view is shown in Fig. 1 ▸. The compound consists of a complex dication, which is in association with four free water mol­ecules and an uncoordinated terephthalate dianion, where the asymmetric unit contains half of these qu­anti­ties. The Ni^2+^ centre is situated on an inversion centre and coordinates to two axial pyridine-3-carbo­nitrile ligands and four equatorial water mol­ecules forming the cationic complex [Ni(H_2_O)_4_(3-CNpy)_2_]^2+^. The bond angles in the cationic part suggest that the complex contains an Ni^2+^ ion in an approximately octa­hedral coordination environment [*cis* angles in the range of 88.66 (4)–91.33 (4)°]. The free terephthalate anion is also located on an inversion centre and has an angle of 14.54 (7)° between the planes of the aromatic ring and of the carboxyl­ate group. Furthermore, it does not coordinate to the Ni^2+^ ion and remains fully deprotonated for charge balance. It also acts as a secondary acceptor to the cationic complex unit. The Ni—O bond lengths are 2.0381 (11) and 2.0519 (9) Å and are in agreement with similar complexes reported (Xiao *et al.*, 2003 ▸; Ma & Xu, 2010 ▸; Ju *et al.*, 2016 ▸). The Ni—N bond length of 2.1481 (11) Å is slightly longer than those in the similar complexes reported by Zukerman-Schpector *et al.* (2000 ▸) and Heine *et al.* (2018 ▸).

## Supra­molecular features   

**Figure 2:**
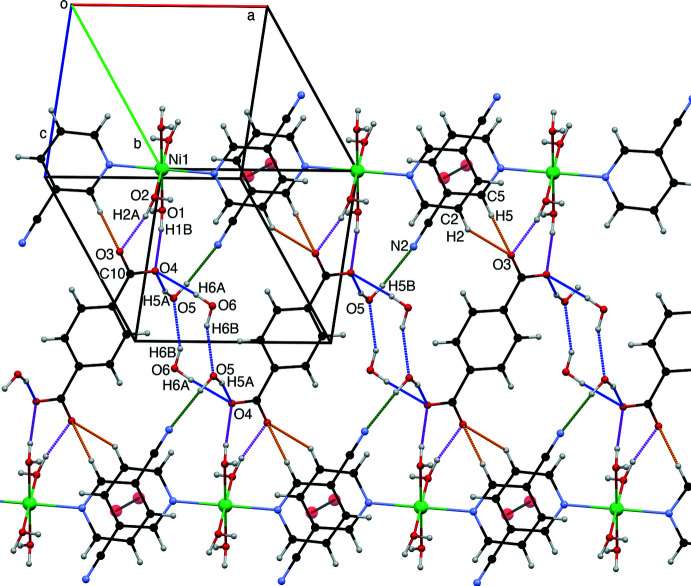
Packing structure of the complex showing the hydrogen bonds and C—H⋯O and π–π inter­actions.

**Figure 3:**
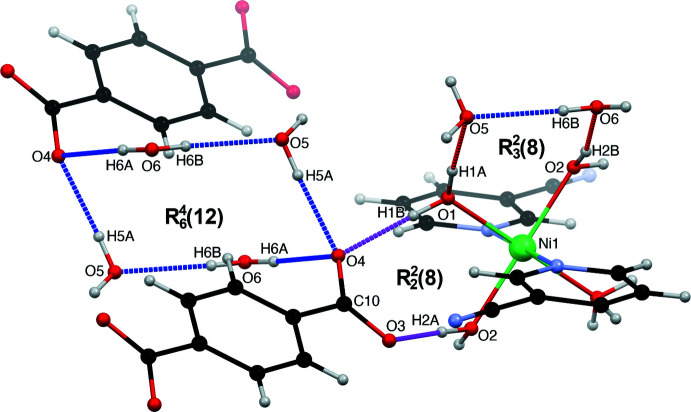
Hydrogen-bonding pattern associated with the 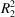
(8), 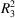
(8) and 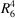
(12) graph sets.

**Figure 4:**
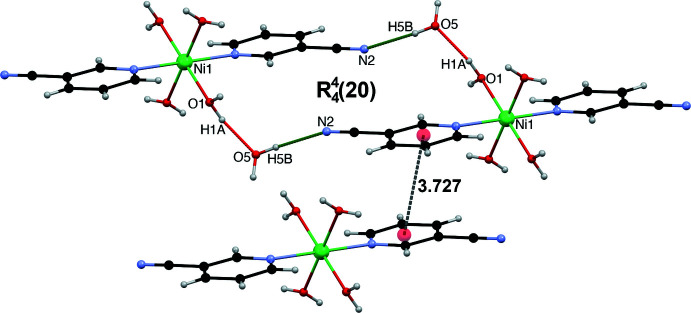
Hydrogen-bonded pattern associated with the 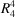
(20) graph set and π–π inter­actions between two 3-NCpy rings.

The supra­molecular structure of the title compound is consolidated by several O—H⋯O and O—H⋯N hydrogen bonds that involve all the possible hydrogen-bond acceptors and donors, which result in the formation of a three-dimensional hydrogen-bonded array in the crystal (Table 1 ▸, Figs. 2 ▸–4 ▸ ▸). The two-dimensional hydrogen-bonded layers featured in Fig. 2 ▸ are connected together *via* hydrogen bonds described by the 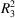
(8) graph set depicted in Fig. 3 ▸. The cationic complex and the terephthalate dianion are hydrogen bonded *via* the O1—H1*B*⋯O4 and O2—H2*A*⋯O3 inter­actions within the 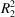
(8) graph set and form infinite chains (Fig. 3 ▸). Neighbouring chains are inter­connected by O1—H1*A*⋯O5 and O5—H5*B*⋯N2 hydrogen bonds described by an 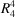
(20) graph set, π–π inter­actions arising from stacking of the 3-NCpy rings [centroid–centroid distance of 3.727 (8) Å, with a slippage of 1.067 Å, Fig. 4 ▸]. The cavity formed is filled by the four solvent water mol­ecules (O5, O6) inter­connecting two neighbouring terephthalate dianions by a cooperative O—H⋯O ring network with an 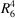
(12) motif, forming infinite chains in a zigzag fashion along the *a*-axis direction (Fig. 3 ▸). Finally, the three-dimensional network is further accomplished among others by the 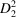
(7) O2—H2*B*⋯O6 hydrogen bonds and C2—H2⋯O3 and C5—H5⋯O3 inter­actions (Table 1 ▸, Fig. 2 ▸). A comprehensive list of first and second level graph sets can be found in Table 2 ▸.

## Database survey   

A survey of the Cambridge Structural Database (CSD version 2020.2; Groom *et al.*, 2016 ▸) for Ni^II^ complexes involving an uncoordinated terephthalate dianion led us to a few results, some of which are as follows. In the complex [Ni(2,2′-bipy)(H_2_O)_4_](C_8_H_4_O_4_) (2,2′-bipy = 2,2′-bipyrid­yl) (CSD refcode: WUWZET) reported by Xiao *et al.* (2003 ▸), the terephthalate anion acts as a synthon to generate a supra­molecular network. The hydrogen bonds between the terephthalate anions and the [Ni(2,2′-bipy)(H_2_O)_4_]^2+^ cations produce a two-dimensional hydrogen-bonded architecture with double sheets. A similar compound, tetra­aqua­bis­(di­methyl­formamide)­nickel(II) tetra­chloro­terephthalate, (QAMDUF; Ma & Xu, 2010 ▸) has a nearly ideal octa­hedral structure with the metal ion lying on an inversion center along with an uncomplexed and fully deprotonated terephthalate dianion. Another Ni^II^–terephthalate complex (AJUPEC; Ju *et al.*, 2016 ▸) with 4,7-di(4-pyrid­yl)-2,1,3-benzo­thia­diazole as auxiliary ligand crystallizes in the monoclinic *P*2_1_/*c* space group. The terephthalate dianion remains uncoordinated and the Ni^II^ ion sits in the centre of an octa­hedron constituted by two pyridyl N atoms in the apical positions and four water oxygen atoms constructing the equatorial plane. The independent cationic units are held together by π–π stacking inter­actions and O—H⋯O hydrogen bonding, generating a compact packing structure. A pyrazine-based Ni^II^–terephthalate complex (AGIWOC; Groeneman & Atwood, 2000 ▸) is a one-dimensional zigzag coordination polymer, where each nickel centre has two *cis μ*-pyrazine ligands along with four coordinated water mol­ecules, giving rise to a distorted octa­hedral coordination environment. A survey of Ni^II^ complexes involving pyridine-3-carbo­nitrile as ligand led us to some other related structures. Heine *et al.* (2018 ▸) investigated the ability of pyridine-3-carbo­nitrile to act as a mono- or bidentate ligand in complexes of the type [*M* ^II^Br_2_(3-CNpy)_*x*_]_*n*_ with *M* ^II^ = Mn, Fe, Co, Ni and *x* = 1, 2 and 4, (CSD refcodes XOSNUR, XOSPAZ, XOSPAZ02) and found that the pyridine-3-carbo­nitrile ligand acted as bridging ligand in complexes with a metal:ligand ratio of 1:1 and as a terminal ligand with ratios of 1:2 and 1:4. In an adduct of Ni^II^ acetyl­acetonate chelating with pyridine-3-carbo­nitrile (MASTUV; Zukerman-Schpector *et al.*, 2000 ▸), the Ni^II^ atom is situated on a centre of symmetry and is octa­hedrally bonded to two equatorial AcAc groups and two pyridine-3-carbo­nitrile groups, which are axially coordinated in a *trans* configuration.

## Synthesis and crystallization   

All reagents were purchased from E. Merck and used without further purification. A mixture of nickel(II) sulfate hepta­hydrate, NiSO_4_·7H_2_O (1.120 g, 4 mmol) and disodium terephthalate, Na_2_C_8_H_4_O_4_ (0.840 g, 4 mmol) was dissolved in 20 mL of water in a 100 mL round-bottomed flask. To this, 0.832 g (8 mmol) of pyridine-3-carbo­nitrile was added and the resulting reaction mixture was stirred mechanically for 2 h. A light-green precipitate was formed. It was filtered, washed with water under suction and dried in a vacuum desiccator over fused CaCl_2_. Green prism-shaped single crystals of the title compound suitable for X-ray diffraction studies were obtained from the undisturbed aqueous reaction solutions after 24 h, yield 73% (1.675 g). The compound is air stable and insoluble in common organic solvents. The crystals remained indefinitely stable against dehydration under ambient conditions. IR spectroscopic data (KBr disc, cm^−1^): *ν*_asym_(OCO^−^) 1568, *ν*_sym_(OCO^−^) 1365, *ν*(C=N) 1602, *ν*(CN_py_) 2243, *δ*_asym_(OCO^−^) 810, *δ*_sym_(OCO^−^) 748. Decomposition point 270°C.

## Refinement   

Crystal data, data collection and structure refinement details are summarized in Table 3 ▸. The non-hydrogen atoms were refined with anisotropic displacement parameters. C-bound hydrogen atoms were placed in idealized positions with C—H = 0.95–0.99 Å, and refined as riding with *U*_iso_(H) = 1.2*U*_eq_(C) or 1.5*U*_eq_(C-meth­yl).

## Supplementary Material

Crystal structure: contains datablock(s) I. DOI: 10.1107/S2056989020015832/jq2002sup1.cif

Structure factors: contains datablock(s) I. DOI: 10.1107/S2056989020015832/jq2002Isup2.hkl

CCDC reference: 1538307

Additional supporting information:  crystallographic information; 3D view; checkCIF report

## Figures and Tables

**Table 1:** Hydrogen-bond geometry (Å, °)

*D*—H⋯*A*	*D*—H	H⋯*A*	*D*⋯*A*	*D*—H⋯*A*
O2—H2*A*⋯O3^i^	0.84 (1)	1.80 (1)	2.6301 (14)	171 (2)
C5—H5⋯O3^i^	0.93	2.46	3.1832 (17)	135
O1—H1*A*⋯O5^ii^	0.83 (1)	1.94 (1)	2.7591 (17)	171 (2)
O2—H2*B*⋯O6^iii^	0.83 (1)	1.86 (1)	2.6710 (17)	168 (2)
O1—H1*B*⋯O4^iv^	0.84 (1)	1.87 (1)	2.7050 (14)	176 (2)
O6—H6*A*⋯O4^v^	0.82 (1)	2.01 (1)	2.8240 (17)	174 (2)
O6—H6*B*⋯O5^vi^	0.84 (1)	2.07 (1)	2.855 (2)	156 (2)
O5—H5*A*⋯O4^v^	0.83 (1)	1.95 (1)	2.7645 (17)	167 (2)
O5—H5*B*⋯N2^vii^	0.83 (1)	2.12 (1)	2.950 (2)	176 (2)

Symmetry codes: (i) 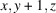; (ii) 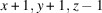; (iii) 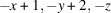; (iv) 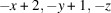; (v) 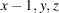; (vi) 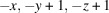; (vii) 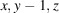.

**Table 2:** Graph-set descriptions

Graph set	Level	Period	No. of Mol­ecules
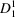 (2) *a*	1		2
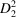 (7) <*a*>*a*	1		3
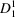 (2) *b*	1		2
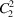 (13) >*b*<*b*	1	2	3
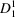 (2) *c*	1		2
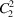 (13) >*c*<*c*	1	2	3
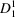 (2) *d*	1		2
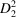 (7) <*d*>*d*	1		3
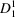 (2) *e*	1		2
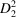 (10) >*e*<*e*	1		3
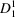 (2) *f*	1		2
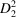 (13) >*f*<*f*	1		3
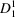 (2) *g*	1		2
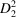 (10) >*g*<*g*	1		3
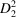 (2) *h*	1		2
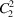 (10) >*a*>*f*	2	2	3
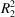 (8) >*b*<*c*	2	2	2
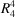 (20) >*a*>f>*a*>*f*	2	2	4

*a* = H1*A*⋯O5, *b* = H1*B*⋯O4, *c* = H2*A*⋯O3, *d* = H2*B*⋯O6, *e* = H5*A*⋯O4, *f* = H5*B*⋯N2, *g* = H6*A*⋯O4 and *h* = H6*B*⋯O5.

**Table 3:** Experimental details

Crystal data	
Chemical formula	[Ni(C_6_H_4_N_2_)_2_(H_2_O)_4_](C_8_H_4_O_4_)·4H_2_O
*M*_r_	575.17
Crystal system, space group	Triclinic, *P* 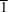
Temperature (K)	293
*a*, *b*, *c* (Å)	8.5709 (17), 8.6760 (17), 9.2644 (19)
α, β, γ (°)	77.26 (3), 81.99 (3), 77.34 (3)
*V* (Å^3^)	652.6 (3)
*Z*	1
Radiation type	Mo *K*α
μ (mm^−1^)	0.81
Crystal size (mm)	0.34 × 0.32 × 0.19

Data collection	
Diffractometer	Bruker APEXII CCD
Absorption correction	Multi-scan (*SADABS*; Sheldrick, 2016 ▸)
*T*_min_, *T*_max_	0.752, 0.826
No. of measured, independent and observed [*I* > 2σ(*I*)] reflections	12067, 3762, 3635
*R*_int_	0.026
(sin θ/λ)_max_ (Å^−1^)	0.704

Refinement	
*R*[*F* ^2^ > 2σ(*F* ^2^)], *wR*(*F* ^2^), *S*	0.026, 0.074, 1.05
No. of reflections	3762
No. of parameters	194
No. of restraints	14
H-atom treatment	H atoms treated by a mixture of independent and constrained refinement
Δρ_max_, Δρ_min_ (e Å^−3^)	0.33, −0.40

Computer programs: *APEX* and *SAINT* (Bruker, 2004 ▸), *SHELXT2014/5* (Sheldrick, 2015*a* ▸), *SHELXL2017/1* (Sheldrick, 2015*b* ▸), *ORTEP-3 for Windows* (Farrugia, 2012 ▸), *Mercury* (Macrae *et al.*, 2020 ▸) and *publCIF* (Westrip, 2010 ▸).

## supplementary crystallographic information

### Crystal data

**Table:**

[Ni(C_6_H_4_N_2_)_2_(H_2_O)_4_](C_8_H_4_O_4_)·4H_2_O	*Z* = 1
*M**_r_* = 575.17	*F*(000) = 300
Triclinic, *P*1	*D*_x_ = 1.463 Mg m^−^^3^
*a* = 8.5709 (17) Å	Mo *K*α radiation, λ = 0.71073 Å
*b* = 8.6760 (17) Å	Cell parameters from 4157 reflections
*c* = 9.2644 (19) Å	θ = 3.4–28.3°
α = 77.26 (3)°	µ = 0.81 mm^−^^1^
β = 81.99 (3)°	*T* = 293 K
γ = 77.34 (3)°	Prism, green
*V* = 652.6 (3) Å^3^	0.34 × 0.32 × 0.19 mm

### Data collection

**Table:**

Bruker APEXII CCD diffractometer	3762 independent reflections
Radiation source: fine-focus sealed tube	3635 reflections with *I* > 2σ(*I*)
Graphite monochromator	*R*_int_ = 0.026
Detector resolution: 8.333 pixels mm^-1^	θ_max_ = 30.0°, θ_min_ = 2.3°
phi and ω scans	*h* = −11→12
Absorption correction: multi-scan (SADABS; Sheldrick, 2016)	*k* = −12→12
*T*_min_ = 0.752, *T*_max_ = 0.826	*l* = −12→12
12067 measured reflections	

### Refinement

**Table:**

Refinement on *F*^2^	Secondary atom site location: difference Fourier map
Least-squares matrix: full	Hydrogen site location: mixed
*R*[*F*^2^ > 2σ(*F*^2^)] = 0.026	H atoms treated by a mixture of independent and constrained refinement
*wR*(*F*^2^) = 0.074	*w* = 1/[σ^2^(*F*_o_^2^) + (0.0422*P*)^2^ + 0.1177*P*] where *P* = (*F*_o_^2^ + 2*F*_c_^2^)/3
*S* = 1.05	(Δ/σ)_max_ < 0.001
3762 reflections	Δρ_max_ = 0.33 e Å^−^^3^
194 parameters	Δρ_min_ = −0.40 e Å^−^^3^
14 restraints	Extinction correction: SHELXL2017/1 (Sheldrick 2015b), Fc^*^=kFc[1+0.001xFc^2^λ^3^/sin(2θ)]^-1/4^
Primary atom site location: structure-invariant direct methods	Extinction coefficient: 0.213 (7)

### Special details

**Table:**

Geometry. All esds (except the esd in the dihedral angle between two l.s. planes) are estimated using the full covariance matrix. The cell esds are taken into account individually in the estimation of esds in distances, angles and torsion angles; correlations between esds in cell parameters are only used when they are defined by crystal symmetry. An approximate (isotropic) treatment of cell esds is used for estimating esds involving l.s. planes.

### Fractional atomic coordinates and isotropic or equivalent isotropic displacement parameters (Å^2^)

**Table:**

	*x*	*y*	*z*	*U*_iso_*/*U*_eq_	
Ni1	1.000000	1.000000	0.000000	0.02537 (8)	
O1	0.98267 (11)	0.96951 (11)	−0.21023 (9)	0.03464 (18)	
H1A	0.990 (2)	1.0424 (15)	−0.2842 (15)	0.052*	
H1B	1.025 (2)	0.8825 (13)	−0.2376 (19)	0.052*	
O2	0.85605 (10)	1.22212 (10)	−0.04411 (9)	0.03425 (17)	
H2A	0.8004 (19)	1.262 (2)	0.0256 (14)	0.051*	
H2B	0.894 (2)	1.2951 (17)	−0.1021 (16)	0.051*	
O3	0.66032 (11)	0.32360 (13)	0.17543 (10)	0.0441 (2)	
O4	0.86703 (10)	0.30492 (11)	0.30301 (10)	0.0407 (2)	
O5	0.03827 (14)	0.21002 (14)	0.54999 (11)	0.0508 (2)	
H5A	−0.026 (2)	0.237 (3)	0.4851 (19)	0.076*	
H5B	0.1233 (16)	0.160 (3)	0.514 (2)	0.076*	
O6	0.03774 (18)	0.55747 (17)	0.26293 (14)	0.0633 (3)	
H6A	−0.011 (3)	0.484 (2)	0.268 (3)	0.095*	
H6B	−0.006 (3)	0.610 (3)	0.328 (2)	0.095*	
N1	0.78988 (11)	0.89668 (11)	0.07505 (11)	0.03167 (19)	
N2	0.33024 (18)	1.0277 (3)	0.4103 (2)	0.0778 (5)	
C1	0.75634 (16)	0.78588 (15)	0.00979 (14)	0.0390 (2)	
H1	0.832785	0.744160	−0.060450	0.047*	
C2	0.61331 (19)	0.73082 (19)	0.04213 (17)	0.0493 (3)	
H2	0.594395	0.654339	−0.006152	0.059*	
C3	0.49961 (17)	0.79021 (19)	0.14618 (17)	0.0483 (3)	
H3	0.401786	0.756363	0.168663	0.058*	
C4	0.53433 (14)	0.90164 (16)	0.21666 (14)	0.0394 (3)	
C5	0.68038 (13)	0.95226 (15)	0.17811 (13)	0.0350 (2)	
H5	0.702508	1.027604	0.225918	0.042*	
C6	0.42063 (16)	0.9712 (2)	0.32545 (18)	0.0533 (4)	
C7	0.44154 (14)	0.43845 (16)	0.39715 (14)	0.0383 (3)	
H7	0.401890	0.397181	0.328307	0.046*	
C8	0.60607 (13)	0.42491 (13)	0.39660 (12)	0.0322 (2)	
C9	0.66387 (14)	0.48662 (16)	0.49989 (14)	0.0388 (3)	
H9	0.773958	0.477732	0.500156	0.047*	
C10	0.71845 (14)	0.34553 (13)	0.28278 (13)	0.0334 (2)	

### Atomic displacement parameters (Å^2^)

**Table:**

	*U*^11^	*U*^22^	*U*^33^	*U*^12^	*U*^13^	*U*^23^
Ni1	0.02417 (11)	0.02844 (11)	0.02502 (11)	−0.00512 (7)	0.00326 (6)	−0.01197 (7)
O1	0.0414 (4)	0.0360 (4)	0.0276 (4)	−0.0048 (3)	0.0005 (3)	−0.0138 (3)
O2	0.0352 (4)	0.0318 (4)	0.0329 (4)	−0.0020 (3)	0.0064 (3)	−0.0108 (3)
O3	0.0389 (5)	0.0569 (6)	0.0407 (5)	−0.0020 (4)	0.0028 (3)	−0.0297 (4)
O4	0.0332 (4)	0.0468 (5)	0.0435 (5)	0.0005 (3)	0.0029 (3)	−0.0242 (4)
O5	0.0513 (6)	0.0591 (6)	0.0366 (5)	−0.0033 (5)	−0.0014 (4)	−0.0071 (4)
O6	0.0782 (8)	0.0595 (7)	0.0548 (7)	−0.0331 (6)	0.0061 (6)	−0.0049 (5)
N1	0.0283 (4)	0.0326 (4)	0.0354 (5)	−0.0073 (3)	0.0000 (3)	−0.0097 (4)
N2	0.0392 (7)	0.1149 (15)	0.0757 (10)	−0.0070 (8)	0.0116 (7)	−0.0295 (10)
C1	0.0431 (6)	0.0373 (6)	0.0400 (6)	−0.0124 (5)	−0.0033 (5)	−0.0110 (5)
C2	0.0564 (8)	0.0489 (7)	0.0515 (8)	−0.0271 (6)	−0.0113 (6)	−0.0084 (6)
C3	0.0382 (6)	0.0576 (8)	0.0513 (7)	−0.0239 (6)	−0.0087 (5)	0.0017 (6)
C4	0.0263 (5)	0.0468 (6)	0.0406 (6)	−0.0077 (4)	−0.0013 (4)	0.0004 (5)
C5	0.0281 (5)	0.0376 (5)	0.0397 (6)	−0.0077 (4)	0.0019 (4)	−0.0105 (4)
C6	0.0286 (6)	0.0724 (10)	0.0542 (8)	−0.0096 (6)	0.0030 (5)	−0.0075 (7)
C7	0.0347 (6)	0.0467 (6)	0.0391 (6)	−0.0053 (5)	0.0019 (4)	−0.0262 (5)
C8	0.0324 (5)	0.0323 (5)	0.0323 (5)	−0.0020 (4)	0.0044 (4)	−0.0162 (4)
C9	0.0297 (5)	0.0487 (7)	0.0414 (6)	−0.0035 (5)	0.0031 (4)	−0.0245 (5)
C10	0.0348 (5)	0.0312 (5)	0.0341 (5)	−0.0033 (4)	0.0071 (4)	−0.0160 (4)

### Geometric parameters (Å, º)

**Table:**

Ni1—O2^i^	2.0381 (11)	N1—C1	1.3409 (15)
Ni1—O2	2.0381 (11)	N2—C6	1.136 (2)
Ni1—O1^i^	2.0519 (9)	C1—C2	1.3828 (19)
Ni1—O1	2.0519 (9)	C1—H1	0.9300
Ni1—N1^i^	2.1481 (11)	C2—C3	1.372 (2)
Ni1—N1	2.1481 (11)	C2—H2	0.9300
O1—H1A	0.829 (9)	C3—C4	1.381 (2)
O1—H1B	0.839 (9)	C3—H3	0.9300
O2—H2A	0.838 (9)	C4—C5	1.3909 (16)
O2—H2B	0.828 (9)	C4—C6	1.438 (2)
O3—C10	1.2396 (15)	C5—H5	0.9300
O4—C10	1.2735 (15)	C7—C9^ii^	1.3873 (16)
O5—H5A	0.830 (9)	C7—C8	1.3885 (17)
O5—H5B	0.830 (9)	C7—H7	0.9300
O6—H6A	0.822 (9)	C8—C9	1.3864 (17)
O6—H6B	0.837 (9)	C8—C10	1.5035 (15)
N1—C5	1.3342 (15)	C9—H9	0.9300
			
O2^i^—Ni1—O2	180.00 (5)	N1—C1—H1	118.5
O2^i^—Ni1—O1^i^	89.76 (5)	C2—C1—H1	118.5
O2—Ni1—O1^i^	90.24 (5)	C3—C2—C1	119.30 (13)
O2^i^—Ni1—O1	90.24 (5)	C3—C2—H2	120.4
O2—Ni1—O1	89.76 (5)	C1—C2—H2	120.4
O1^i^—Ni1—O1	180.0	C2—C3—C4	118.21 (12)
O2^i^—Ni1—N1^i^	89.33 (4)	C2—C3—H3	120.9
O2—Ni1—N1^i^	90.67 (4)	C4—C3—H3	120.9
O1^i^—Ni1—N1^i^	88.66 (4)	C3—C4—C5	119.42 (12)
O1—Ni1—N1^i^	91.33 (4)	C3—C4—C6	121.56 (12)
O2^i^—Ni1—N1	90.67 (4)	C5—C4—C6	118.98 (13)
O2—Ni1—N1	89.33 (4)	N1—C5—C4	122.40 (12)
O1^i^—Ni1—N1	91.33 (4)	N1—C5—H5	118.8
O1—Ni1—N1	88.67 (4)	C4—C5—H5	118.8
N1^i^—Ni1—N1	180.0	N2—C6—C4	179.2 (2)
Ni1—O1—H1A	122.0 (12)	C9^ii^—C7—C8	120.18 (11)
Ni1—O1—H1B	121.0 (12)	C9^ii^—C7—H7	119.9
H1A—O1—H1B	106.7 (15)	C8—C7—H7	119.9
Ni1—O2—H2A	120.0 (12)	C9—C8—C7	119.43 (10)
Ni1—O2—H2B	118.1 (12)	C9—C8—C10	121.09 (10)
H2A—O2—H2B	107.6 (15)	C7—C8—C10	119.47 (10)
H5A—O5—H5B	107.4 (17)	C8—C9—C7^ii^	120.39 (11)
H6A—O6—H6B	107.9 (18)	C8—C9—H9	119.8
C5—N1—C1	117.63 (10)	C7^ii^—C9—H9	119.8
C5—N1—Ni1	120.87 (8)	O3—C10—O4	124.25 (10)
C1—N1—Ni1	121.17 (8)	O3—C10—C8	117.83 (11)
N1—C1—C2	123.01 (13)	O4—C10—C8	117.92 (10)

Symmetry codes: (i) −*x*+2, −*y*+2, −*z*; (ii) −*x*+1, −*y*+1, −*z*+1.

### Hydrogen-bond geometry (Å, º)

**Table:**

*D*—H···*A*	*D*—H	H···*A*	*D*···*A*	*D*—H···*A*
O2—H2*A*···O3^iii^	0.84 (1)	1.80 (1)	2.6301 (14)	171 (2)
C5—H5···O3^iii^	0.93	2.46	3.1832 (17)	135
O1—H1*A*···O5^iv^	0.83 (1)	1.94 (1)	2.7591 (17)	171 (2)
O2—H2*B*···O6^v^	0.83 (1)	1.86 (1)	2.6710 (17)	168 (2)
O1—H1*B*···O4^vi^	0.84 (1)	1.87 (1)	2.7050 (14)	176 (2)
O6—H6*A*···O4^vii^	0.82 (1)	2.01 (1)	2.8240 (17)	174 (2)
O6—H6*B*···O5^viii^	0.84 (1)	2.07 (1)	2.855 (2)	156 (2)
O5—H5*A*···O4^vii^	0.83 (1)	1.95 (1)	2.7645 (17)	167 (2)
O5—H5*B*···N2^ix^	0.83 (1)	2.12 (1)	2.950 (2)	176 (2)

Symmetry codes: (iii) *x*, *y*+1, *z*; (iv) *x*+1, *y*+1, *z*−1; (v) −*x*+1, −*y*+2, −*z*; (vi) −*x*+2, −*y*+1, −*z*; (vii) *x*−1, *y*, *z*; (viii) −*x*, −*y*+1, −*z*+1; (ix) *x*, *y*−1, *z*.
